# Comparison between amniotomy, oxytocin or both for augmentation of labor in prolonged latent phase: a randomized controlled trial

**DOI:** 10.1186/1477-7827-8-136

**Published:** 2010-11-07

**Authors:** Zohar Nachum, Gali Garmi, Yfat Kadan, Noah Zafran, Eliezer Shalev, Raed Salim

**Affiliations:** 1 Rappaport Faculty of Medicine Technion, Israel Institute of Technology, Department of Obstetrics and Gynecology, Ha'Emek Medical Center, Afula, Israel

## Abstract

**Background:**

A prolonged latent phase is independently associated with an increased incidence of subsequent labor abnormalities. We aimed to compare between oxytocin augmentation, amniotomy and a combination of both on the duration of labor among women with a prolonged latent phase.

**Methods:**

Women with a singleton fetus in cephalic presentation who have a prolonged latent phase, were randomly allocated to amniotomy (group 1), oxytocin (group 2) or both (group 3). A group of women who progressed spontaneously without intervention composed the control group (group 4). The primary outcome was the duration of time from initiation of augmentation until delivery.

**Results:**

A total of 213 women were consented and randomized to group 1 (70 women), group 2 (72 women) and group 3 (71 women). Group 4 was composed from additional 70 women. A mean reduction of 120 minutes in labor duration was observed among group 3 compared to group 1 (p = 0.08) and 180 minutes compared to group 2 and 4 (p = 0.001). Women in group 3 had a shorter length of time from augmentation until the beginning of the active phase and a shorter first stage of labor than group 1 (p = 0.03), group 2 (p = 0.001) and group 4 (p = 0.001). Satisfaction was greater among group 3 and 4. Mode of delivery and neonatal outcome were comparable between the groups.

**Conclusion:**

Labor augmentation by combined amniotomy and oxytocin among women with a prolonged latent phase at term seems superior compared to either of them alone.

## Background

Arrested or prolonged labor is a frequent indication for cesarean delivery [1,2]. Prolonged labor is also associated with increased pain and negative birth experiences [3,4]. Furthermore, women with a longer first stage of labor have experienced a higher rate of postpartum hemorrhage, chorioamnionitis and neonatal admission to the intensive care unit [5].

A prolonged latent phase is independently associated with an increased incidence of subsequent labor abnormalities, an increased rate of cesarean delivery, thick meconium, depressed Apgar scores, and the need for newborn resuscitation [6,7].

The incidence of a prolonged latent phase has been reported to be three to four percent regardless of parity [8]. Although the optimal management is uncertain, augmentation of labor has been proposed as an adequate approach to the problem of prolonged latent phase, as well as a strategy to reduce the rate of cesarean delivery [7]. This intervention is based on the hypothesis that the most frequent cause of dystocia is inadequate uterine contraction.

Oxytocin augmentation of uterine contractions with or without amniotomy is widely used in the modern obstetric practice to treat a slow labor, although the timing of oxytocin initiation and amniotomy may vary widely [9].

Our aim in this study was to compare among women with a prolonged latent phase, the effect on the duration of labor, of augmentation with oxytocin, amniotomy or a combination of both.

## Methods

This prospective randomized trial was held from January 2006 to January 2009 in the labor and delivery ward of the department of Obstetrics and Gynecology at Ha'Emek Medical Center in Afula, Israel, a university teaching hospital.

Women at term (gestational age of 37 weeks or more) with intact amniotic membranes and a singleton fetus in cephalic presentation, who had a spontaneous onset of labor, a cervical dilatation between two and four cm, vertex level of no more than two cm above the pelvic inlet and had a prolonged latent phase of labor were included in the study. Gestational age was confirmed by either a documented first trimester ultrasound or by known regular last menstrual period and a documented ultrasound in the second trimester.

The latent phase was defined as the interval between the start of regular contractions (women's report) combined with any cervical dynamics (dilatation and/or effacement) until the active phase of labor was established when cervical dilatation was greater than four cm. A prolonged latent phase was defined as lasting more than 20 hours for primiparous women and 14 hours for multiparous women [10].

Women with a previous uterine scar, rupture of membranes, placental abruption, severe preeclampsia, suspected fetal macrosomia (greater than 4000 g), a non-reassuring fetal heart rate tracing or any contraindication for a trial of labor, were excluded. Women who had a malformed fetus diagnosed in the antepartum period or had an antepartum fetal death were also excluded.

Women with the diagnosis of prolonged latent phase were randomly allocated to amniotomy (group 1), oxytocin (group 2) or both (group 3). Amniotomy in group 1 was performed immediately after admission to the delivery ward. If no progress in cervical dilatation was documented after two hours or there were fewer than three contractions in ten minutes recorded after one hour, oxytocin was added. In group 2, oxytocin was started immediately after admission to the delivery ward. If no progress in cervical dilatation was documented after two hours or there were fewer than three contractions in ten minutes recorded after 1 hour, amniotomy was performed. Group 3 had both amniotomy and oxytocin performed and started simultaneously after admission to the delivery ward. The protocol of oxytocin administration was one mU/min increased by one mU/min every 20 minutes until five contractions in ten minutes or cervical progress was documented. When no progress was documented after two hours despite five contractions in ten minutes, an internal pressure catheter was inserted and oxytocin was increased until a Montevideo score of 200 to 300 was achieved.

Randomization of the three groups was performed in blocks of ten using a computer randomization sequence generation program. The sequence was concealed until intervention was assigned.

Women who were admitted in the latent phase of labor to our labor ward and who progressed spontaneously without intervention composed the control group (group 4). The control group was selected by choosing the women who met the inclusion criteria during the study period. The purpose of selecting this group of women is to compare the duration of the different labor stages among the study groups to a spontaneously progressing women who did not need labor augmentation.

The primary outcome was the duration of time from initiation of labor augmentation until delivery. Women who were operated were excluded from the analysis of the primary outcome. Secondary outcomes were active phase duration, duration of first and second stages of labor, mode of delivery, maternal fever, antibiotic administration, postpartum hemorrhage, anal sphincter tears, Apgar score and maternal satisfaction.

Women expressed their satisfaction using a score from one (absolutely not satisfied) to five (absolutely satisfied). The following variables were considered: maternal age, ethnicity, parity, antepartum obstetric complications (gestational diabetes, hypertension, antepartum bleeding, thrombophilia, and oligohydramnios), epidural use, number of vaginal examinations, intra-partum fetal scalp-electrode or uterine pressure catheter use and birth weight.

Continuous electronic fetal heart rate monitoring for all groups was initiated and continued until delivery. Arrest of dilatation during the active phase was determined when the Montevideo score was between 200 and 300 for more than two hours. Arrest of descent was defined as an arrest of descent of the fetal head for more than one hour during the second stage of labor. When arrest of descent was diagnosed at a level below the midpelvis, a vacuum extraction was used, and when above the midpelvis, a cesarean delivery was performed. All groups were given standard care according to departmental guidelines when it came to midwifery support, i.e. 1 midwife per 1 to 2 laboring women and access to pain relief, i.e. epidural, intravenous pethidine and/or inhalation of a mixture of O2 with N2O according to maternal demand regardless of the measured cervical dilatation.

The study was approved by the local institutional review board and each woman signed an informed consent.

### Statistical analysis

One way ANOVA or a Kruskal Wallis test in the case of non-normally distributed variables was performed to compare the continuous data of the four groups of women. Chi-square or Fisher exact tests when appropriate were used to compare the categorical data. Bonferroni post hoc tests in the case of normally distributed data or Mann-Whitney pair-wise comparisons with p < 0.05 considered to be significant. In the case of non-normally distributed data, Kruskal-Wallis test was used to determine pair-wise differences among statistically significant and borderline statistically significant variables. This analysis was repeated for primiparous and for multiparous women.

Mean time interval from initiation of augmentation until delivery was eight hours (+120 minutes), based on previous observation at our delivery ward. Accordingly, in order to demonstrate a difference of one hour between group three compared to group one and two with an alpha of 0.05 and a power of 90%, a sample size of 63 women per group was required. The primary analysis was performed on the groups as allocated, that is, by the intention to treat, including all women as randomized.

## Results

**Figure 1:**
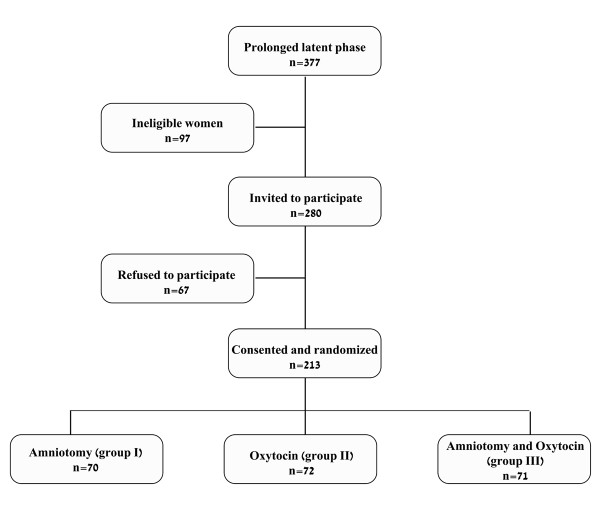
**Flow diagram of participants**.

Of 12,571 women who delivered during the study period, 377 (3%) had a prolonged latent phase. After excluding 97 ineligible women, 280 women fulfilled the inclusion criteria and were invited to participate in the study. Of them 213 consented and were randomized (Figure 1). Seventy women composed the control group (group 4).

The four groups were demographically similar and had no statistically significant differences in pre-delivery parameters (table 1). Length of time from augmentation until delivery was significantly different between the 3 study groups. Post hoc testing revealed that women in group 3 had a statistically shorter labor than groups 2 and 4 (p = 0.001) and tended to have a shorter labor than group 1 (p = 0.08).

**Table 1:** Demographic and obstetric characteristics of all women by treatment group

	Group 1 (Amniotomy)	Group 2 (Oxytocin)	Group 3 (Amniotomy and Oxytocin)	Group 4 (Control)	
Obstetric and demographic data	N = 70	N = 72	N = 71	N = 70	p
Maternal age, years	28.1 ± 4.9 (28)	28.5 ± 5.3 (27)	28.2 ± 5.0 (28)	28.7 ± 4.7 (29)	0.9
Gestational age, weeks	39.6 ± 1.1 (39.6)	39.6 ± 1.2 (39.6)	39.6 ± 1.1 (40)	39.7 ± 1.0 (39.6)	0.9
Parity	2.5 ± 1.3 (2)	2.5 ± 1.3 (2)	2.6 ± 1.4 (2)	2.2 ± 1.2 (2)	0.2
BMI (pre pregnancy) kg/m^2^	24.6 ± 4.8 (23)	23.3 ± 3.5 (23)	25.4 ± 5.6 (24)	23.5 ± 4.1 (23)	0.09
Weight gain, kg	12.2 ± 5.7 (12)	13.0 ± 5.6 (13)	12.5 ± 5.1 (12)	13.6 ± 5.1 (13)	0.5
Cervical dilatation, cm	3.0 ± 0.4 (3)	2.8 ± 0.4 (3)	3.0 ± 0.4 (3)	3.0 ± 1.0 (3)	0.3
Intra-partum fever	3 (4)	0 (0.0)	0 (0.0)	0 (0.0)	0.03
Antibiotics use	4 (6)	0 (0.0)	0 (0.0)	0 (0.0)	0.007
Epidural use	18 (26)	23 (32)	18 (25)	21 (30)	0.9
Fetal scalp pH	1 (1)	0 (0.0)	0 (0.0)	0 (0.0)	0.5
Vaginal examinations	6.0 ± 2.8 (5)^a^	6.1 ± 2.4 (6)^a^	4.8 ± 1.6 (5)	5.6 ± 2.0 (6)	0.02
Fetal Scalp electrode	10 (14)	3 (4)	12 (17) ^c^	15 (21) ^c^	0.02
Intra-uterine pressure catheter	4 (6)	2 (3)	0 (0.0)	1 (1)	0.1
Time from enrollment until delivery, min	431 ± 346 (320)	494 ± 327 (396)^a^	312 ± 245 (246)	498 ± 306 (390)^a^	0.001
Duration of the first stage of labor, min	403 ± 327 (273)^a^	463 ± 313 (387)^a^	283 ± 233 (222)	460 ± 285 (371)^a^	0.001
Time from enrollment until the beginning of the active phase, min	284 ± 296 (180)^a^	361 ± 303 (278)^a^	183 ± 222 (109)	333 ± 270 (265)^a^	0.001
Duration of the active phase, min	118 ± 115 (85)	103 ± 89 (78)	101 ± 65 (89)	127 ± 96 (101)	0.3
Duration of the second stage, min	32 ± 51 (10)	31 ± 46 (10)	28 ± 41 (10)	38 ± 54 (12)	0.7
Delivery mode					0.8
Spontaneous vaginal	65 (93)	68 (94)	66 (93)	68 (97)	----
Vacuum	3 (4)	2 (3)	4 (6)	1 (1)	------
Cesarean	2 (3)	2 (3)	1 (1)	1 (1)	0.9
Vacuum or cesarean	5 (7)	4 (6)	5 (7)	2 (3)	0.6
Early post partum hemorrhage	0 (0.0)	3 (4)	3 (4)	0 (0.0)	0.09
Anal sphincter tear	0 (0.0)	0 (0.0)	0 (0.0)	0 (0.0)	1.0
Post-partum fever	1 (1)	0 (0.0)	0 (0.0)	0 (0.0)	0.5
Women satisfaction	4.7 ± 0.6 ^b^	4.7 ± 0.6^b^	4.9 ± 0.5^b^	5.0 ± 0.1	0.001
Birthweight, gr	3371 ± 424 (3377)	3386 ± 434 (3350)	3419 ± 409 (3376)	3309 ± 385 (3289)	0.5
Apgar score at 5 min	10.0 ± 0.2	10.0 ± 0.3	9.9 ± 0.2	10.0 ± 0.3	0.9

Data are mean ± standard deviation (median) or n (%) unless otherwise specified.

a, statistical difference (p < 0.05) was found when compared with group 3 after post hoc testing;

b, statistical difference (p < 0.05) was found when compared with group 4 after post hoc testing.

c, statistical difference (p < 0.05) was found when compared with group 2 after post hoc testing.

The four groups differed significantly in the length of time of the first stage and the length of time from augmentation until the beginning of the active phase (p = 0.001). Post hoc testing revealed that women in group 3 had a shorter first stage of labor than group 1 (p = 0.03), group 2 (p = 0.001) and group 4 (p = 0.001). This difference was also found in the length of time from augmentation until the beginning of the active phase.

The four groups differed in the frequency of intrapartum fever (p = 0.03) and in antibiotic use (table 1); however post hoc pairwise comparisons did not find any significant differences between the groups in the frequency of intrapartum fever and in antibiotic use.

The four groups differed in the number of vaginal examinations (p = 0.02). Post hoc testing revealed that the women in group 3 had fewer vaginal examinations than either of the single augmentation groups (group 1: p = 0.02; group 2: p = 0.007) but did not differ from the control group.

There was a statistically significant difference in women satisfaction among the four groups (p = 0.01). Post hoc testing showed that the control group was more satisfied than groups 1, 2 (p = 0.001) and 3 (p = 0.02). In addition group 3 was more satisfied than group 2 (p = 0.007).

### Primiparous women

Of all women recruited, 80 were primiparous (table 2). The four groups were demographically similar. There were no statistically significant differences in pre-delivery parameters between the groups.

**Table 2:** Demographic and obstetric characteristics of primiparous women by treatment group

	Group 1 (Amniotomy)	Group 2 (Oxytocin)	Group 3 (Amniotomy and Oxytocin)	Group 4 (Control)	
Obstetric and demographic data	N = 21	N = 20	N = 16	N = 23	p
Intra-partum fever	3 (14)	0 (0.0)	0 (0.0)	0 (0.0)	0.03
Antibiotics use	3 (14)	0 (0.0)	0 (0.0)	0 (0.0)	0.03
Epidural use	12 (57)	8 (40)	7 (44)	11 (48)	0.7
Fetal scalp pH	1 (5)	0 (0.0)	0 (0.0)	0 (0.0)	0.7
Vaginal examinations	8.2 ± 3.5 (7)^a^	6.4 ± 1.8 (6)	5.1 ± 1.2 (5)	6.7 ± 2.0 (7)	0.009
Fetal Scalp electrode	7(33)	2 (10)	4 (25)	7 (30)	0.3
Intra-uterine pressure catheter	4 (19)	0 (0.0)	0 (0.0)	0 (0.0)	0.008
Time from enrollment until delivery, min	629 ± 338 (545)^a^	590 ± 367 (444)	420 ± 319 (363)	740 ± 294 (689)^a^	0.005
Duration of the first stage of labor, min	555 ± 307 (506)^a^	533 ± 359 (409)^a^	351 ± 327 (253)	651 ± 391 (580)^a^	0.003
Time from enrollment until the beginning of the active phase, min	339 ± 349 (298)^a^	426 ± 372 (303)^a^	206 ± 298 (105)	435 ± 305 (370)^a^	0.009
Duration of the active phase, min	216 ± 161 (153)	114 ± 54 (119)^b^	139 ± 84 (140)	205 ± 104 (178)	0.01
Duration of the second stage, min	75 ± 64 (51)	57 ± 65 (32)	69 ± 49 (50)	89 ± 66 (86)	0.4
Delivery mode					0.5
Spontaneous vaginal	16 (76)	17 (85)	14 (88)	22 (96)	----
Vacuum	3 (14)	2 (10)	2 (13)	1 (4)	---
Cesarean	2 (10)	1 (5)	0 (0)	0 (0)	0.4
Vacuum or cesarean	5 (24)	3 (15)	2 (13)	1 (4)	0.3
Early post partum hemorrhage	0 (0.0)	1 (5)	0 (0.0)	0 (0.0)	0.5
Post-partum fever	0 (0.0)	0 (0.0)	0 (0.0)	0 (0.0)	----
Women satisfaction	4.4 ± 0.9 (5)	4.4 ± 0.7 (4)^b^	4.8 ± 0.5 (5)	5.0 ± 0.2 (5)	0.002
Birthweight, gr	3266 ± 487 (3334)	3257 ± 297 (3314)	3480 ± 385 (3428)	3337 ± 424 (3298)	0.4
Apgar score at 5 min	10.0 ± 0.2	10.0 ± 0.2	9.9 ± 0.2	9.9 ± 0.4	1.0

Data are mean + standard deviation (median) or n (%) unless otherwise specified.

a, statistical difference (p < 0.05) was found when compared with group 3 after post hoc testing;

b, statistical difference (p < 0.05) was found when compared with group 4 after post hoc testing.

Length of time from augmentation until delivery was significantly different between the groups among primiparous women (p = 0.005). Post hoc testing revealed that women in group 3 had a statistically shorter labor than group 4 (p = 0.001) and 1 (p = 0.04), and tended to have a shorter labor than group 2 (p = 0.08).

The four groups differed significantly in the length of time of the first stage (p = 0.003), the length of time from augmentation until the beginning of the active phase (p = 0.009) and the active phase duration (p = 0.02). Post hoc testing revealed that women in group 3 had a shorter first stage of labor than the control group (p = 0.001), group 2 (p = 0.01) and group 1 (p = 0.02). This difference was also found in the length of time from augmentation until the beginning of the active phase (control: p = 0.004; group 1: p = 0.01; group 2: p = 0.003). Post hoc testing of the active phase differences revealed that the active phase was shorter among group 2 than the control group (p = 0.002).

The four groups differed in the frequency of intrapartum fever (p = 0.03) and in antibiotic use; however post hoc pairwise testing did not find any significant differences between any of the augmentation methods. The four groups differed in the number of vaginal examinations (p = 0.009). Post hoc testing revealed that the women in group 3 had less vaginal examinations than women in group 1 (p = 0.001). Finally, there was a statistically significant difference in women satisfaction among the four groups (p = 0.002). Post hoc testing showed that the women in group 4 were more satisfied than women in groups 1 and 2 (group 1: p = 0.04; group 2: p = 0.01) yet not different than the women in group 3.

### Multiparous women

Of all women recruited, 203 were multiparous (table 3). The four groups were demographically similar. There were no statistically significant differences in the pre-delivery variables between the groups.

**Table 3:** Demographic and obstetric characteristics of multiparous women by treatment group

	Group 1 (Amniotomy)	Group 2 (Oxytocin)	Group 3 (Amniotomy and Oxytocin)	Group 4 (Control)	
Obstetric and demographic data	N = 49	N = 52	N = 55	N = 47	p
Intra-partum fever	0 (0.0)	0 (0.0)	0 (0.0)	0 (0.0)	---
Antibiotics use	1 (2)	0 (0.0)	0 (0.0)	0 (0.0)	0.5
Epidural use	6 (12)	15 (29)	11 (20)	10 (21)	0.2
Fetal scalp pH	0 (0.0)	0 (0.0)	0 (0.0)	0 (0.0)	-----
Vaginal examinations	5.0 ± 1.7 (5)	6.0 ± 2.6 (5)	4.8 ± 1.7 (5)	5.0 ± 1.7 (5.0)	0.1
Fetal Scalp electrode	3 (6)	1 (2)	8 (15)^d^	8 (17)^d^	0.03
Intra-uterine pressure catheter	0 (0.0)	2 (4)	0 (0.0)	1 (2)	0.3
Time from enrollment until delivery, min	352 ± 320 (241)	457 ± 305 (384)^a,c^	279 ± 210 (227)	376 ± 232 (345)^a^	0.001
Duration of the first stage of labor, min	337 ± 316 (230)	436 ± 292 (379)^a^	262 ± 197 (214)	364 ± 230 (332)^a^	0.001
Time from enrollment until the beginning of the active phase, min	260 ± 313 (164)	336 ± 272 (276)^a^	176 ± 197 (109)	279 ± 237 (233)^a^	0.001
Duration of the active phase, min	76 ± 49 (70)	99 ± 99 (65)	90 ± 55 (81)	87 ± 61(75)	0.6
Duration of the second stage, min	15 ± 31 (8)	21 ± 32 (9)	16 ± 29 (10)	13 ± 15 (7)	0.5
Delivery mode					0.4
Spontaneous vaginal	49 (100)	51(98)	52 (95)	46 (98)	----
Vacuum	0 (0)	0 (0)	2 (4)	0 (0)	----
Cesarean	0 (0)	1 (2)	1 (2)	1 (2)	0.9
Vacuum or cesarean	0 (0)	1 (2)	3 (6)	1 (2)	0.4
Early post partum hemorrhage	0 (0.0)	2 (4)	3 (6)	0 (0.0)	0.2
Post-partum fever	1 (2.0)	0 (0.0)	0 (0.0)	0 (0.0)	0.5
Women satisfaction	4.9 ± 0.4 (5)^b^	4.8 ± 0.5 (5)^b^	4.9 ± 0.5 (5)	5.0 ± 0.0 (5)	0.01
Birthweight, gr	3416 ± 391 (3396)	3435 ± 470 (3456)	3401 ± 417 (3376)	3296 ± 368 (3282)	0.4
Apgar score at 5 min	10.0 ± 0.1	10.0 ± 0.3	10.0 ± 0.2	10.0 ± 0.2	0.8

Data are mean + standard deviation (median) or n (%) unless otherwise specified.

a, statistical difference (p < 0.05) was found when compared with group 3 after post hoc testing;

b, statistical difference (p < 0.05) was found when compared with group 4 after post hoc testing;

c, statistical difference (p < 0.05) was found when compared with group 1 after post hoc testing;

d, statistical difference (p < 0.05) was found when compared with group 2 after post hoc testing.

Length of time from augmentation until delivery was significantly different between the groups for multiparous women (p = 0.001). Post hoc testing revealed that women in group 3 had a significantly shorter labor than groups 2 and 4 (p = 0.001, p = 0.004) yet not significantly different than group 1. Group 1 had a significantly shorter labor than group 2 (p = 0.008).

The four groups significantly differed in the length of time of the first stage (p = 0.001) and the length of time from augmentation until the beginning of the active phase (p = 0.001). Post hoc testing revealed that women in group 3 had a shorter first stage of labor than group 2 (p = 0.001) and group 4 (p = 0.004). This difference was also found in the length of time from augmentation until the beginning of the active phase (group 2: p = 0.001, group 4: p = 0.003). Women in group 3 had no difference in the length of first stage of labor or the length of time from augmentation until the beginning of the active phase than group 1.

The four groups differed in the frequency of usage of a scalp electrode (p = 0.03). Women in group 2 required a scalp electrode less often than the women in group 3 (p = 0.03) or 4 (p = 0.01).

There was a statistically significant difference in women satisfaction among the four groups (p = 0.01). Post hoc testing showed that the control group was more satisfied than group 1 and 2 (group 1: p = 0.007; group 2: p = 0.001), yet not different than the women in group 3.

## Discussion

The incidence of prolonged latent phase has been reported to be three to four percent regardless of parity [8]. While Friedman did not find that prolongation of the latent phase adversely influence maternal or fetal outcome [11], others reported an increased incidence of subsequent labor abnormalities, increased rate of cesarean delivery, thick meconium and depressed newborn [6,7]. The optimal management of a prolonged latent phase is uncertain and several authors have called for further studies by means of randomized controlled trials [7].

Since inefficient uterine contractions is the most common cause of poor progress [12], we compared, in this randomized controlled trial between amniotomy, oxytocin or a combination of both among women with a prolonged latent phase. We investigated the effect of each intervention on the duration of labor and on maternal and neonatal outcomes. Our results indicated that combined oxytocin and amniotomy resulted in a shorter labor duration compared to either amniotomy or oxytocin alone. A mean reduction of 120 and 180 minutes in labor duration when both methods of interventions were used was observed compared to amniotomy alone or oxytocin alone respectively. The main impact was observed from the initiation of augmentation until the beginning of the active phase. The effect on the duration of labor was evident among the whole group as well as in both primiparous and multiparous women.

Mode of delivery did not differ between the groups, and was comparable to the mode of delivery among a control group of women who progressed spontaneously without intervention. Post partum complications and neonatal outcome were also comparable. The overall low rate of cesarean section and post partum complications observed in this study is probably due to the fact that low risk women were primarily included other than them having a prolonged latent phase.

The mean number of vaginal examinations was lower among women in the combined augmentation group compared to either of them alone. This observation is probably attributed to the shorter length of labor duration. Both the shorter duration of labor and fewer vaginal examinations probably contributed to a greater satisfaction observed among both primiparous and multiparous women. Moreover, when both amniotomy and oxytocin were used, labor duration was shorter and women satisfaction and neonatal outcome were similar to a control group of women who progressed spontaneously without intervention. Since, there is no physiologic difference between oxytocin-stimulated labor and natural labor [13], shorter labor among women who have augmentation of labor with both oxytocin and amniotomy may be explained by probably an exposure to a higher overall dosage of oxytocin compared to spontaneously progressing women. This observation is supported by studies that compared between low versus high dose oxytocin regimens for augmentation of labor and which reported that a higher dosage regimen was associated with a significantly shorter labors [13,14].

Contradictory results have been reported regarding the effect of augmentation of labor on the length of labor duration, mode of delivery and neonatal outcome [15-19]. Our results regarding shortening of labor duration and a lack of difference in the operative delivery rate between the groups corresponds well to earlier conclusions [16-18].

Oxytocin augmentation of uterine contractions combined with or without amniotomy is widely used to treat slow labor, although the exact timing of initiation and their combination varies widely. Beside, intervention is not always without risks. Intervention with oxytocin has been associated with uterine hyperstimulation and fetal heart rate abnormalities [20], while amniotomy was associated with increased infectious morbidity [21]. The common practice of including amniotomy as a complement to oxytocin for labor augmentation could mask the benefits as well as the risks of each intervention [16-18,22]. The strength of this study is that we were able to isolate the impact of early oxytocin administration compared to amniotomy or both as the main contrasts between the groups. Furthermore, we added a fourth group of normally progressing women to check the duration of a spontaneously progressing labor and to compare it with the length of labor among the study groups. The comparison may assist physicians when counseling women regarding labor duration before augmentation is attempted. Although other maternal and neonatal outcomes were comparable between the groups, the study was not powered to detect outcomes other than the duration of labor.

## Conclusion

Augmentation of the contractile effort is mandated to enable labor to progress to a normal vaginal delivery among women with prolonged latent phase. In this situation, combined oxytocin and amniotomy seems superior compared to either of them alone.

## Competing interests

The authors declare that they have no competing interests.

## Authors' contributions

ZN and RS conceived and designed the study, supervised the data collection, assisted in the analysis and drafted the manuscript. GG, YK and NZ participated in the design of the study, assisted in collection and maintenance of the data and co-wrote the article. ES assisted in conceiving, designing and analysis, and edited the manuscript. All authors read and approved the final manuscript.

## Authors' information

ZN is a lecturer, Rappaport Faculty of Medicine Technion, Israel Institute of Technology, senior consultant obstetrician, Department of Obstetrics and Gynecology Ha'Emek Medical Center, Afula, Israel; GG, YK and NZ are residents, Department of Obstetrics and Gynecology Ha'Emek Medical Center, Afula, Israel; ES is a professor and associate Dean, Rappaport Faculty of Medicine Technion, Israel Institute of Technology, Chairman, Department of Obstetrics and Gynecology Ha'Emek Medical Center, Afula, Israel and RS is a lecturer, Rappaport Faculty of Medicine Technion, Israel Institute of Technology, senior obstetrician, head of delivery ward, Department of Obstetrics and Gynecology Ha'Emek Medical Center, Afula, Israel.
